# Dual EZH2 and EHMT2 histone methyltransferase inhibition increases biological efficacy in breast cancer cells

**DOI:** 10.1186/s13148-015-0118-9

**Published:** 2015-08-21

**Authors:** Edward Curry, Ian Green, Nadine Chapman-Rothe, Elham Shamsaei, Sarah Kandil, Fanny L Cherblanc, Luke Payne, Emma Bell, Thota Ganesh, Nitipol Srimongkolpithak, Joachim Caron, Fengling Li, Anthony G. Uren, James P. Snyder, Masoud Vedadi, Matthew J. Fuchter, Robert Brown

**Affiliations:** Department of Surgery and Cancer, Ovarian Cancer Action Research Centre, Imperial College London, Hammersmith Hospital Campus, London, W12 ONN UK; Division of Cancer, Department of Surgery and Cancer, Imperial College London, Hammersmith Hospital Campus, London, W12 ONN UK; Department of Chemistry, Imperial College London, South Kensington Campus, London, SW7 2AZ UK; Department of Pharmacology, Emory University, Atlanta, GA 30322 USA; Structural Genomics Consortium, University of Toronto, Toronto, ON M5G 1L7 Canada; MRC Clinical Sciences Centre, Hammersmith Hospital Campus, London, W12 0NN UK; Department of Chemistry, Emory University, Atlanta, GA 30322 USA; Section of Molecular Pathology, Institute of Cancer Research, Sutton, SM2 5NG UK

## Abstract

**Background:**

Many cancers show aberrant silencing of gene expression and overexpression of histone methyltransferases. The histone methyltransferases (HKMT) EZH2 and EHMT2 maintain the repressive chromatin histone methylation marks H3K27me and H3K9me, respectively, which are associated with transcriptional silencing. Although selective HKMT inhibitors reduce levels of individual repressive marks, removal of H3K27me3 by specific EZH2 inhibitors, for instance, may not be sufficient for inducing the expression of genes with multiple repressive marks.

**Results:**

We report that gene expression and inhibition of triple negative breast cancer cell growth (MDA-MB-231) are markedly increased when targeting both EZH2 and EHMT2, either by siRNA knockdown or pharmacological inhibition, rather than either enzyme independently. Indeed, expression of certain genes is only induced upon dual inhibition. We sought to identify compounds which showed evidence of dual EZH2 and EHMT2 inhibition. Using a cell-based assay, based on the substrate competitive EHMT2 inhibitor BIX01294, we have identified proof-of-concept compounds that induce re-expression of a subset of genes consistent with dual HKMT inhibition. Chromatin immunoprecipitation verified a decrease in silencing marks and an increase in permissive marks at the promoter and transcription start site of re-expressed genes, while Western analysis showed reduction in global levels of H3K27me3 and H3K9me3. The compounds inhibit growth in a panel of breast cancer and lymphoma cell lines with low to sub-micromolar IC50s. Biochemically, the compounds are substrate competitive inhibitors against both EZH2 and EHMT1/2.

**Conclusions:**

We have demonstrated that dual inhibition of EZH2 and EHMT2 is more effective at eliciting biological responses of gene transcription and cancer cell growth inhibition compared to inhibition of single HKMTs, and we report the first dual EZH2-EHMT1/2 substrate competitive inhibitors that are functional in cells.

**Electronic supplementary material:**

The online version of this article (doi:10.1186/s13148-015-0118-9) contains supplementary material, which is available to authorized users.

## Background

EZH2 along with EED and SUZ12 are the indispensible core components of the Polycomb Repressive Complex (PRC2) responsible for maintenance of the repressive epigenetic mark H3K27me3: trimethylation of lysine 27 of histone 3 [1]. High expression of the histone methyltransferase (HKMT) EZH2, in some cases associated with gene amplification, has been well documented in a variety of cancers [2], [3]. EZH2 overexpression has been linked to poor prognosis [4, 5] and shown to be a marker of aggressive breast cancer [6], associated with difficult-to-treat basal or triple negative breast cancer [7]. Gene knockdown of EZH2 reduces growth of a variety of tumour cell types [5, 8, 9]. Several groups have reported specific co-factor competitive EZH2 inhibitors [10–16], which have shown a strong capacity to reduce growth of cells expressing mutated forms of EZH2 (such as certain non-Hodgkin’s lymphoma [12]). However, removal of the repressive mark H3K27me3 alone may not always be sufficient for reversal of gene silencing. Indeed, it has been shown that highly specific EZH2 inhibitors require a mutant EZH2 status to inhibit cell growth, being less effective in cells solely expressing wild type EZH2 [5, 8, 9]. Elimination of further repressive methylation marks by inhibition of additional HKMTs may be required to fully realise the epigenetic potential of HKMT inhibitors.

EHMT2 (also known as G9a) and the highly homologous EHMT1 (also known as GLP) are HKMTs partly responsible for mono- and di-methylation of lysine nine of histone 3 (H3K9me1 and H3K9me2, respectively); repressive chromatin marks found on the promoter regions of genes that are often aberrantly silenced in cancer [17]. EHMT2 is overexpressed and amplified in various cancers including leukaemia, prostate carcinoma, and lung cancer, with gene knockdown of EHMT2 inhibiting cancer cell growth in these tumour types [18, 19]. BIX-01294 (see Fig. 2) was previously identified as an inhibitor of the HKMTs EHMT2 and EHMT1, and subsequent medicinal chemistry studies around the 2, 4-diamino-6, 7-dimethoxyquinazoline template of BIX-01294 have yielded a number of follow-up EHMT2 inhibitors [20–25].

In addition to its role in methylating H3K9, EHMT2 has been shown to be able to methylate H3K27 [26, 27]. It has been suggested that this could provide cells with a mechanism to compensate in part for a loss of EZH2 [28]. The picture is further complicated by recent evidence that EHMT2 and EZH2 (via the PRC2 complex) interact physically and share targets for epigenetic silencing [29]. Combining this evidence, it would again suggest that specifically targeting either EZH2 or EHMT2 alone may not be sufficient to reverse epigenetic silencing of genes, but rather combined inhibition may be required. To this end, we have examined the effect of dual EZH2 and EHMT2 gene knockdown or enzyme inhibition in breast cancer cells. Consistent with the requirement for removal of both repressive H3K9 and H3K27 methylation marks, we show that dual inhibition of EHMT2 and EZH2 pharmacologically or by SiRNA is necessary for reactivation of certain genes and induces greater inhibition of cell growth than targeting either HKMT alone in triple negative breast cancer MDA-MB-231 cells. Further, we have identified proof-of-concept compounds which are dual (substrate competitive) EZH2-EHMT1/2 inhibitors.

## Results

### Combined inhibition of EZH2 and EHMT2 is more effective at inducing gene re-expression and inhibiting tumour cell growth than single HKMT inhibition

**Fig. 1:**
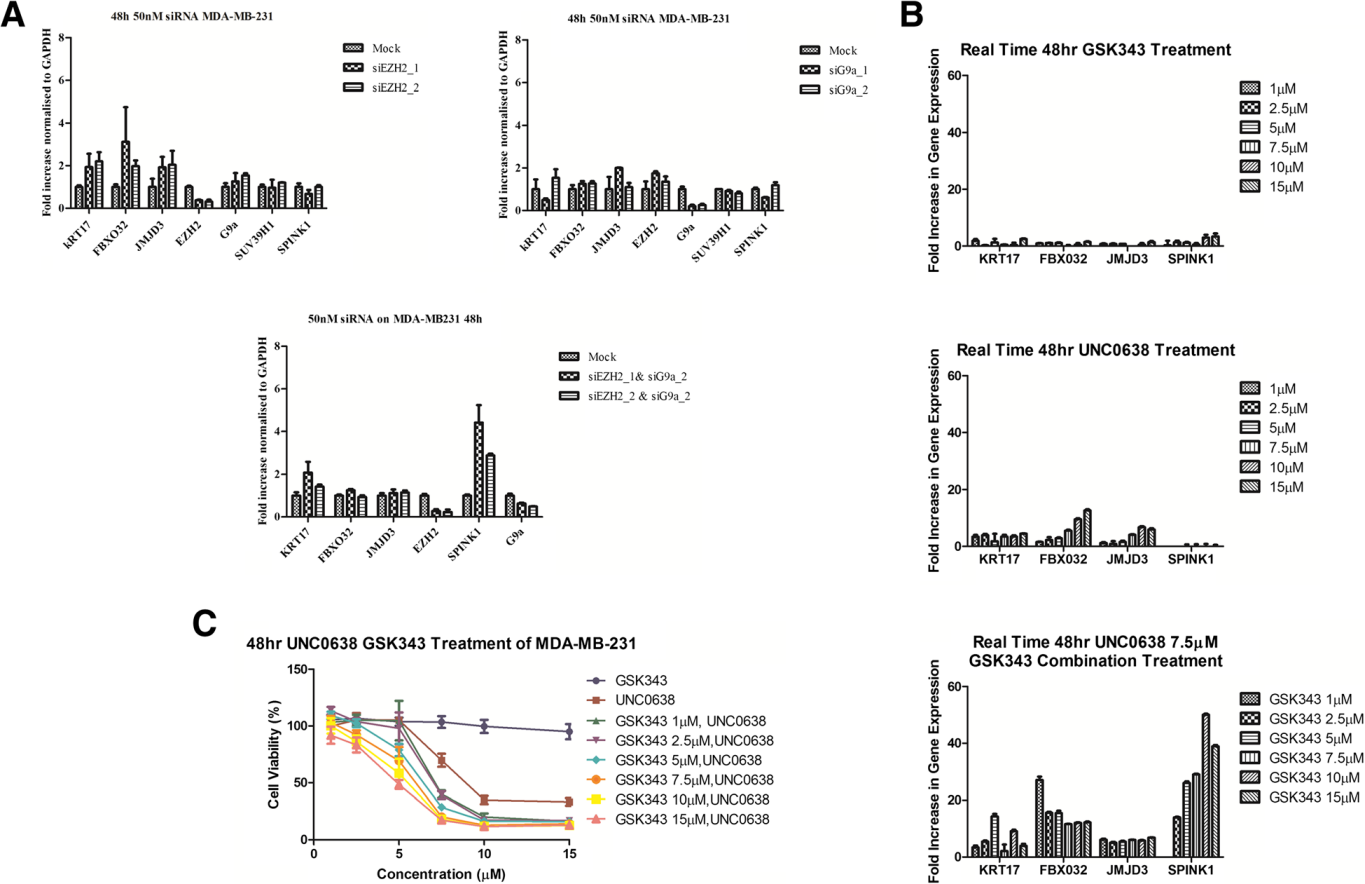
MTT and mRNA levels in MDA-MB-231 cells after pharmacological inhibition and siRNA knockdown of EZH2 and EHMT2(G9a), individually and in combination. **a** Expression levels of KRT17, FBX032, JMJD3, EZH2, SPINK1 and EHMT2 were measured by qRT-PCR in the MDA-MB-231 cell line 48 h after transfection with siRNAs targeting EZH2 and EHMT2, both individually and in combination. All measurements were normalised to the fold-change (relative to GAPDH) in the mock transfection control. Error bars represent the mean ± SD of experiment performed in technical triplicate. **b** Expression levels of KRT17, FBX032, JMJD3 and SPINK1 were measured by qRT-PCR in the MDA-MB-231 cell line treated for 48 h with GSK343, UNC0638, and UNC0638 (at 7.5 μM) with increasing doses of GSK343. Each group has been compared to the untreated sample following normalisation to GAPDH. Error bars represent the mean ± SD of experiment performed in technical triplicate. **c** MTT assay for cell viability of MDA-MB-231 cells after treatment. MDA-MB-231 cells were seeded in 96 well plates. After 24 h, increasing doses of GSK343, UNC0638 or combination treatments (1, 2.5, 5, 7.5, 10 and 15 μM) were added to cells. Control was media with 0.5 % DMSO. Cell viability was measured by MTT assay after a 48-h treatment and a 24-h proliferation period. Error bars represent the mean ± SEM of five independent repeats

SiRNA knockdown in the MDA-MB-231 breast tumour cell line was used to examine the effect of combined inhibition of *EZH2* and *EHMT2* expression on epigenetic regulation at select target genes, compared to knockdown of either gene alone (Fig. 1a). Knockdown of *EZH2* with two independent SiRNAs induced 2–4-fold increased mRNA levels of *KRT17* and *FBXO32*, genes which are known to be silenced in an EZH2 dependent manner [30]. Knockdown of *EHMT2* (G9a) had limited effects on mRNA levels of these target genes. However, double knockdown of *EZH2* and *EHMT2* had dramatic effects on the level of *SPINK1* mRNA, a gene which was not upregulated by silencing of EZH2 or EHMT2 individually. Thus, for at least certain genes, dual reduction in *EZH2* and *EHMT2* levels is necessary to observe marked changes in target gene expression 48 h following knockdown.

**Fig. 2:**
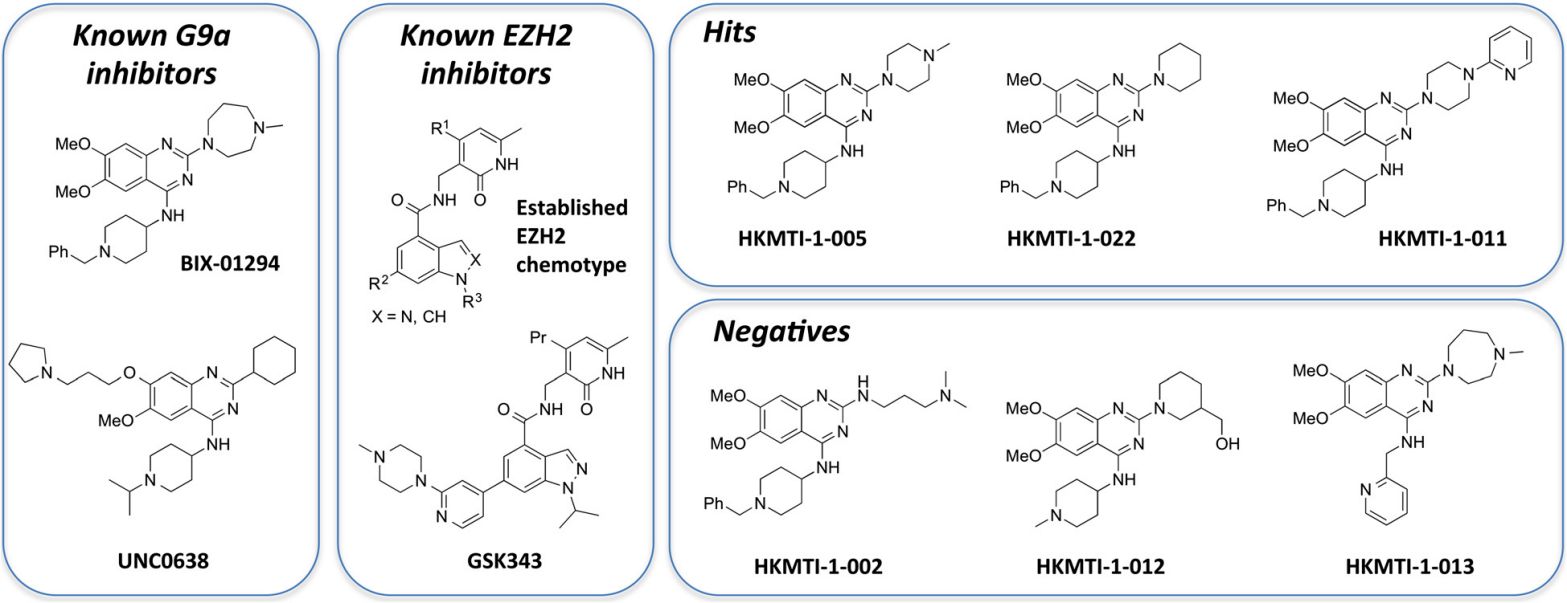
Chemical structure of histone lysine methyltransferase inhibitors

The effects on gene expression of the selective EZH2 inhibitor GSK343 [10] and the selective EHMT2 inhibitor UNC0638 [22] (Fig. 2) used alone or in combination were also examined using the MDA-MB-231 triple negative breast cancer cell line (Fig. 1b). When MDA-MB-231 cells were treated with the EZH2 inhibitor GSK343 at 1–15 μM for 48 h alone, there was little change in the mRNA levels of *KRT17*, *FBX032* and *SPINK1* and the H3K27 demethylase *JMJD3* (Fig. 1b). UNC0638 at 1–15 μM for 48 h alone showed dose-dependent upregulation of *FBX032* and *JMJD3*; however, *KRT17* and *SPINK1* mRNA levels were not significantly altered. However, the combination treatments with GSK343 and UNC0638 showed marked increase in mRNA levels of all the target genes, in contrast to the single agent treatment. Consistent with dual *EZH2*/*EHMT2* SiRNA knockdown, *SPINK1* has the biggest change in mRNA levels between the single and combination treatments, having a 50-fold increase with the combination treatment. Global levels of H3K27me3 and H3K9me3 in MDA-MB-231 cells were examined following treatment with GSK343 and UNC0638 as single agents and in combination (Additional file 1: Figure S1). Together, these data show a minor decrease in global levels of these silencing marks following treatment with either GSK343 or UNC0638 as single agents across a range of doses yet a strong dose-dependent decrease in the levels of these marks when cells are treated with these compounds in combination. This provides further compelling evidence for the efficacy of dual HKMT inhibition in reversal of epigenetic silencing in cancer cells.

Next, the effects on cell viability of GSK343 and UNC0638 used alone or in combination were examined (Fig. 1c). Treatment alone with GSK343 showed no significant reduction in cell viability up to 15 μM, while UNC0638 sole treatment caused a dose dependant reduction in cell viability, with a calculated IC_50_ of 9 μM. When the cells were treated with both compounds in combination, a marked increase in growth inhibition was observed when compared to single agent treatment using UNC0638 or GSK343 (Fig. 1c). This is particularly apparent at a 5 μM concentration of both compounds, where alone they have no significant effect on reducing cell viability, while in combination, they markedly reduce cell viability to >50 % (*p* < 0.01).

### Analogues of an EHMT2-specific inhibitor can upregulate EZH2 silenced genes

Both EZH2 and EHMT1/2 belong to the SET-domain superfamily [31], the catalytic SET-domain being responsible for the methylation of the targeted lysine residues. BIX-01294 has previously been shown, both structurally and biochemically, to bind to the substrate (histone)-binding pocket of EHMT1/2 [32]. Since protein recognition motifs for histone binding at repressive sites are similar [33] and EHMT2 has been shown to be able to methylate H3K27, in addition to its more common H3K9 target [27], it is likely that there are common aspects to the histone substrate binding pockets of the repressive HKMTs EZH2 and EHMT1/2. We therefore felt it would be feasible to use quinazoline template of BIX-01294 in the discovery of dual (substrate competitive) EZH2-EHMT1/2 inhibitors.

A compound library based on the selective BIX-01294 EHMT2 inhibitor was synthesised and characterised analogously to previously reported methods [20–22, 24, 25, 32]. In light of the reported selectivity of this chemical scaffold towards EHMT1/2, the library was primarily examined for compounds showing additional EZH2 inhibitory activity, as defined by re-expression of KRT17 and FBXO32, genes which are known to be silenced in an EZH2 dependent manner [30]. The majority of compounds had little or no effect on both KRT17 and FBXO32 RNA levels. However, we identified three compounds which upregulate KRT17 and FBXO32 RNA levels. The data for these compounds along with a comparison of the related EHMT2 inhibitors BIX-01294 and UNC0638 and a representative number of negative compounds are shown in Table 1 (for chemical structures see Fig. 2 and the Additional file 2 for hit characterisation data). All hit compounds—HKMTI-1-005, HKMTI-1-011, HKMTI-1-022—showed upregulation of KRT17, FBXO32, and JMJD3 mRNA at a 10 μM dose. The reported EHMT2-specific inhibitors BIX-01294 and UNC0638, while being closely related to our hits from a chemical structure perspective, elicit different effects on expression of the target genes. BIX-01294 (Table 1, entry 4) does not upregulate KRT17 but does upregulate FBXO32. This is compatible with the observation that FBXO32 is regulated via multiple mechanisms, potentially responding to a variety of factors [34]. An analogous effect is observed for UNC0638 (Table 1, entry 5). The specific EZH2 inhibitor GSK343 has no effect whatsoever on all the target genes studied (Table 1, entry 6) when examined up to 72 h following treatment and at concentrations up to 10 μM.

**Table 1:** RT-PCR data for a single dose of a panel of HKMT inhibitor compounds

Entry		Compound	KRT17	FBXO32	JMJD3	EZH2
1	Hit	HKMTI-1-005	4.05	3.65	3.12	0.63
2	Hit	HKMTI-1-022	4.28	29.4	11.56	0.21
3	Hit	HKMTI-1-11	6.95	33.25	6.25	0.22
4	G9ai	BIX01294	1.06	3.34	2.7	0.87
5	G9ai	UNC0638^a^	1.1	5.5	3.4	0.4
6	EZH2i	GSK343	0.9	1.2	1.0	1.0
7	Negative	HKMTI-1-002	0.66	1.12	1.57	0.86
8	Negative	HKMTI-1-012	1.32	1.06	0.9	1.38
9	Negative	HKMTI-1-013	0.78	0.93	0.87	0.13

RNA levels for target genes are normalised against the housekeeping gene GAPDH, and shown is the fold increase compared to the mock treated sample

^a^UNC0638 treatment at 7.5 μM, all other compounds given at 10 μM

**Fig. 3:**
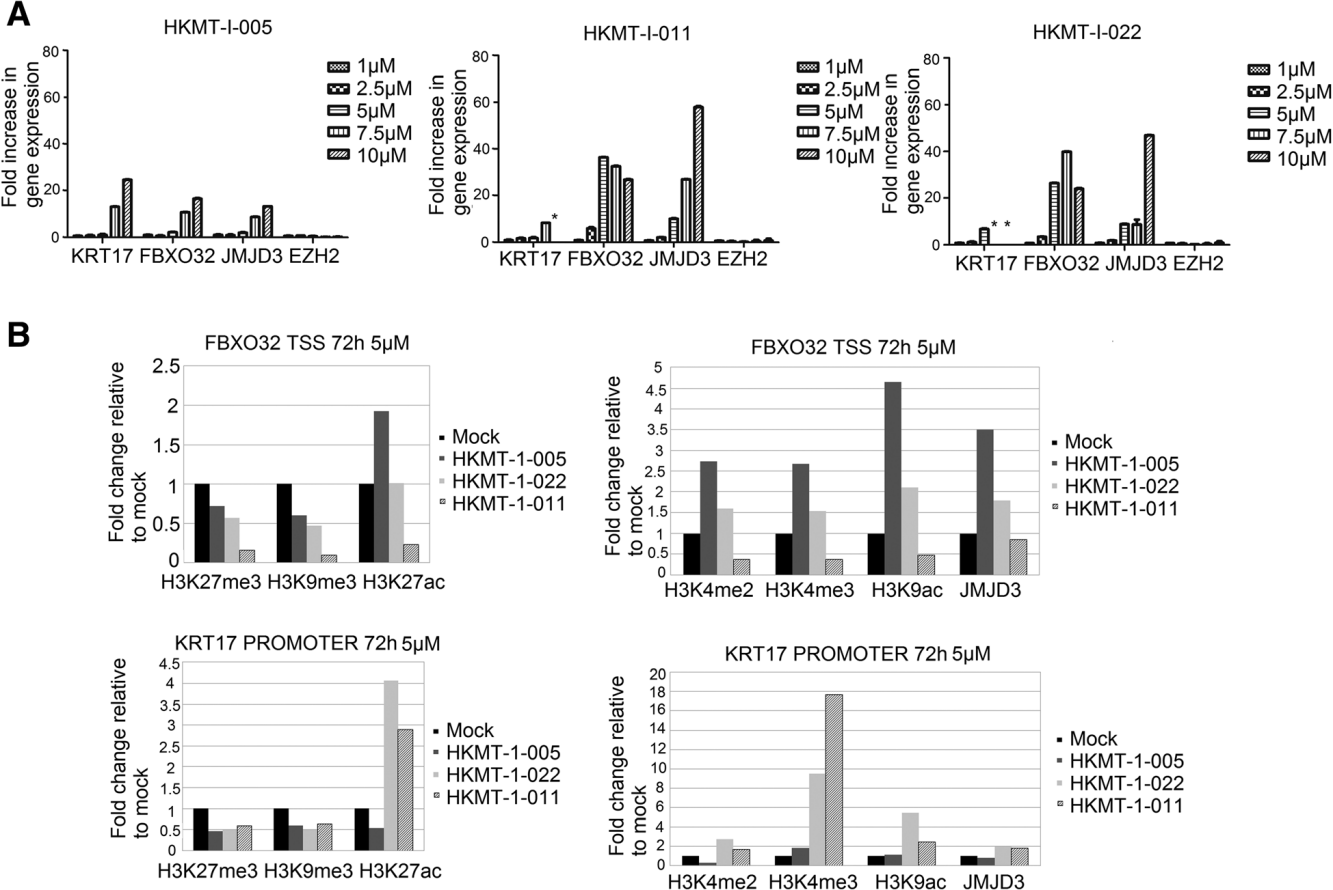
Effects of hit compounds on RNA levels and histone marks. **a** Sybr green real-time PCR mRNA level measurement of EZH2 target genes and executing enzymes following a 48-h compound treatment at different concentrations of MDA-MB-231 cells. Measurements marked with an ‘*’ are below detection limit, most likely due to cell death. All RT-PCR experiments were performed in triplicate, normalised to GAPDH and displayed as fold difference to the untreated sample. Error bars represent the mean ± SD of experiment performed in technical triplicate. **b** Sybr green real-time PCR measurement of the FBXO32 transcription start site and KRT17 promoter region following chromatin immunoprecipitation, using antibodies to the histone marks shown, of MDA-MB-231 cells treated with three selected compounds at 5 μM for 72 h. Shown are representative examples of triplicate ChIP experiments which consistently showed the same changes. The fold difference to the untreated sample is shown. Each IP-value has been determined as the relative increase to the no-antibody control and then normalised to GAPDH levels

To further evaluate the three hit compounds identified, we treated MDA-MB-231 cells for 48 and 72 h at various concentrations of compounds and examined gene expression effects (Fig. 3a). All hit compounds showed a dose-dependent increase of KRT17, FBXO32, as well as JMJD3 mRNA. Higher doses of certain compounds started to cause cell death, and because of this, at these doses, the expression of KRT17 was often below the detection limit of low-expressed genes.

Chromatin immunoprecipitation (ChIP) experiments were carried out on treated MDA-MB-231 cells to verify that the detected gene upregulation is indeed due to chromatin remodelling (Fig. 3b). We tested the silencing marks H3K9me3 and H3K27me3 as well as the activating marks H3K4me3, H3K4me2, H3K27ac and H3K9ac. All three compounds showed a clear decrease in repressive chromatin marks (H3K27me3, H3K9me3), and at least in some instances, an increase in permissive marks, at two target genes (Fig. 3b). This is consistent with the compounds having dual HKMT inhibitory activity in removing both H3K9me and H3K27me marks, while allowing activating marks to be established at these loci.

### Genome-wide changes in gene expression

**Fig. 4:**
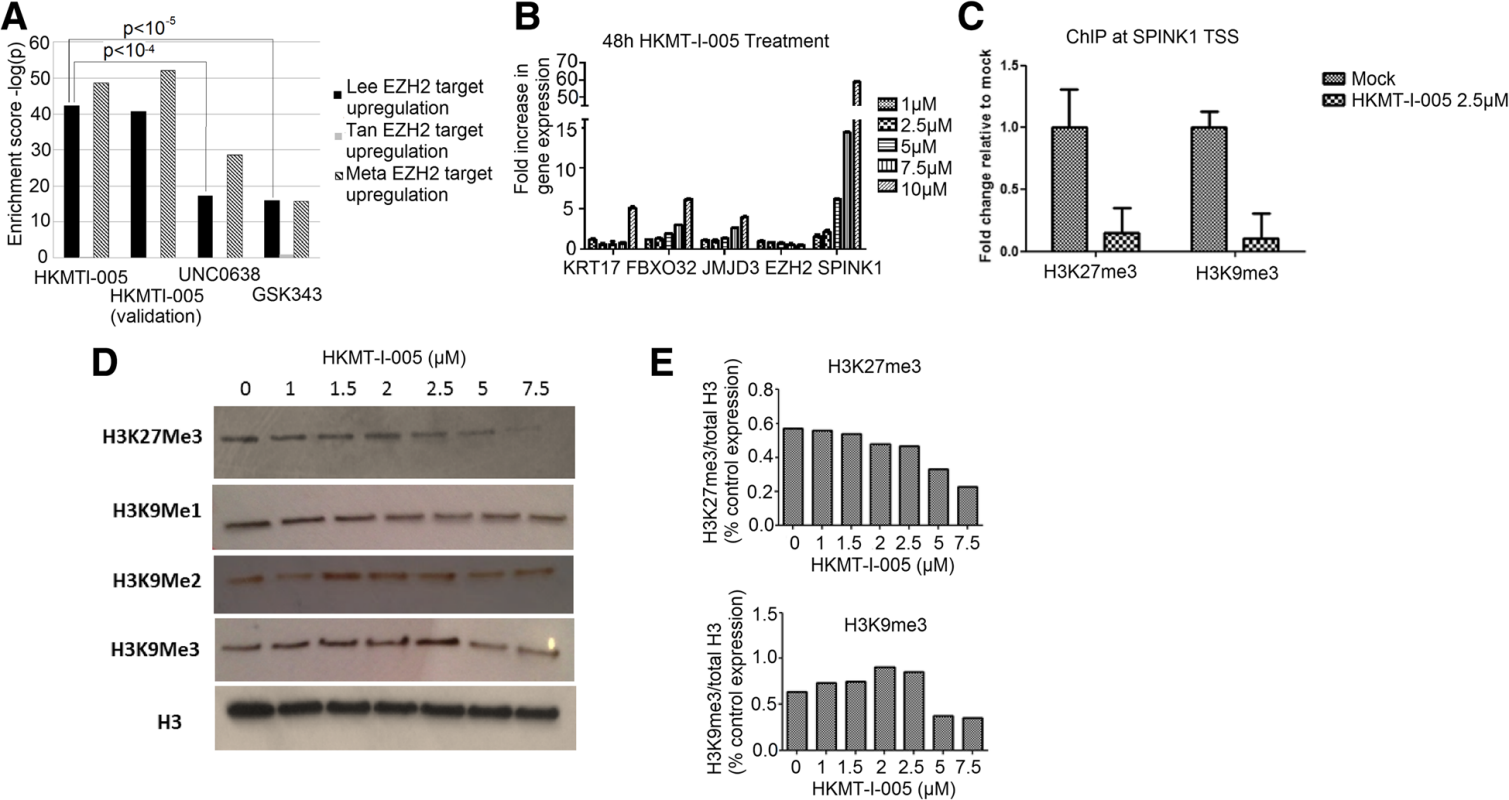
Compound-induced upregulation of EZH2-repressed target genes. **a** Enrichment scores for differential expression of EZH2 targets on treatment with panel of compounds. Enrichment scores are negative logarithm of *p* values, such that higher values indicate more significant enrichment. *Left-hand bars* show enrichment of targets derived from siRNA knockdown of EZH2 in MDA-MB-231 cell line (labelled ‘Lee EZH2 target upregulation’), *middle bars* show enrichment of targets derived from siRNA knockdown of EZH2 in MCF7 cell line (labelled ‘Tan EZH2 target upregulation’) and *right-hand bars* show enrichment of targets defined by meta-analysis of 18 independent microarray studies profiling effects of shRNA-mediated EZH2 knockdown in a variety of cell lines (labelled ‘Meta EZH2 target upregulation’): See ‘Materials and Methods’. **b** Sybr green real-time PCR mRNA level measurement of EZH2 target genes and executing enzymes following a 48-h treatment with HKMTI-1-005 at different concentrations of MDA-MB-231 cells. **c** Sybr green real-time PCR measurement of the SPINK1 transcription start site following chromatin immunoprecipitation, using antibodies to the histone marks shown, of MDA-MB-231 cells treated with HKMT-I-005 or HKMT-I-011 at 2.5 μM for 24 h. Each IP-value has been determined as the relative increase to the no-antibody control and is shown as fold difference relative to the untreated control. **d** Western blot showing levels of modified histones, following a 48-h treatment with HKMTI-1-005 at different doses. Total H3 levels are shown for comparison. **e** Densitometry quantification of Western blot intensity, showing ratio of modified (H3K27me3 top, H3K9me3 bottom) H3 relative to total H3 with increasing dose of HKMTI-1-005 treatment

Agilent microarrays were used to perform gene expression profiling in MDA-MB-231 breast cancer cells after 24 h of treatment with the hit compound HKMTI-1-005, the EZH2 inhibitor GSK343 [10] and EHMT2 inhibitor UNC0638 [22]. To validate the finding of the initial expression data for the hit compounds, a second microarray experiment was performed on the same platform using HKMTI-1-005-treated MDA-MB-231 cells after 24 h of treatment. To assess the extent to which our selected analogues—derived from the selective EHMT1/2 inhibitor BIX-01294—had gained EZH2 inhibitory activity, lists of genes activated or repressed following siRNA knockdown of EZH2 in MDA-MB-231 cells were identified [35] and shown in Additional file 3: Table S4. These lists of target genes were investigated in the context of genome-wide changes in gene expression following treatment with the compounds. HKMTI-1-005 showed very significant enrichment for upregulation of EZH2 silenced genes (Fig. 4a) in both the initial array (*p* = 4.53x10^−43^) and the validation array (*p* = 1.99x10^−49^). GSK343 and UNC0638 also both showed a significant upregulation of EZH2 target genes (Fig. 4a) though to a lesser extent than HKMTI-1-005. Indeed, analysis of the difference in systematic upregulation showed that HKMTI-1-005 upregulated EZH2-silenced genes significantly more than either GSK343 (*p* = 5.8x10^−5^) or UNC0638, (*p* = 1.7x10^−4^).

The same enrichment tests were repeated using target gene sets identified in an EZH2 siRNA knockdown study in another breast cancer cell line, MCF-7 [30]. Almost no enrichment was observed of this gene set in MDA-MB-231 cells after treatment with any of the compounds (HKMTI-1-005, GSK343 and UNC0638) (Fig. 4a), suggesting that EZH2 has cell-type-specific targets. To investigate this further, we undertook a meta-analysis to identify consensus target genes based on 18 independent EZH2 siRNA studies (details of the meta-analysis are provided in ‘Methods’ also see Additional file 4: Table S5). Encouragingly, treatment of MDA-MB-231 cells with HKMTI-1-005 resulted in highly significant upregulation of these consensus EZH2-repressed genes (Fig. 4a). This suggests that key EZH2 target genes that are conserved across a wide range of cell lines are re-expressed upon treatment with our dual HKMT inhibitor. Furthermore, this identifies generally applicable pharmacodynamic biomarkers of EZH2 inhibitors across cell types.

### Compound-induced changes in H3K9me and H3K27me levels in cells

The microarray data showed a clear upregulation of the levels of SPINK1 mRNA (a gene previously identified as a target for dual EZH2 and EHMT2 inhibition, see Fig. 1) following treatment with HKMTI-1-005, an observation that was confirmed via qRT-PCR (Fig. 4b). These qRT-PCR experiments demonstrated a dose-dependent upregulation of SPINK1 alongside a re-evaluation of the candidate genes (KRT17, FBX032, JMJD3) chosen for the initial compound screen. Furthermore, ChIP-PCR at the SPINK1 transcription start site clearly demonstrated a reduction in both H3K27me3 and H3K9me3 in MDA-MB-231 cells after treatment with 2.5 μM HKMT-I-005 (Fig. 4c). More broadly, Western analysis showed that global levels of H3K27me3 and H3K9me3 are reduced in MDA-MB-231 cells after treatment with HKMTI-1-005 (Fig. 4d), and densitometry analysis (Fig. 4e) suggests this happens in dose-dependent manner. Together, these data strongly support that the hit compound HKMT-I-005 reduces levels of H3K27me3 and H3K9me3 at concentrations of compound that are less or equivalent to the growth inhibition IC50 concentration for MDA-MB-231 (Table 2). For comparison, similar analysis of global levels of H3K27me3 and H3K9me3 in MDA-MB-231 cells was performed following treatment with GSK343 and UNC0638 as single agents and in combination (Additional file 1: Figure S1).

**Table 2:** Cell growth IC50 of HKMT inhibitors in MDA-MB-231 cells

Cell growth assay (IC50 μM)	HKMT-1-005	BIX-01294	UNC0638	GSK343
Cell viability (MTT)	4.3	9.6	8.2	>15
Clonogenic	0.41	1.4	1.1	>50

IC50s (μM) for the dual inhibitor compounds HKMT-1-005 and HKMT-1-011 in cell viability (MTT assay) or clonogenic growth assays of MDA-MB-231 cells compared to the starting point for the chemical library BIX-01294, as well as UNC0638 and GSK343

In order to identify specific pathways being transcriptionally modulated, the microarray data was analysed for enrichment of pathways belonging to each pathway listed on the ConsensusPathDB (CPDB) database [36]. The Benjamini-Hochberg-adjusted [37] enrichment *p* value estimates for each treatment is given in Additional file 5: Table S6. Interestingly, genes belonging to the pathway ‘Apoptosis’ displayed a highly significant systematic shift towards upregulation on treatment with our hit compound(s) at 24 h (*p* < 1E-4) but not the selective EZH2 (GSK343) or EHMT2 (UNC0638) inhibitor compound (*p* = 0.42 and *p* = 0.30, respectively). Consistent with induction of apoptosis-related genes, hit compound HKMTI-1-005 induces apoptosis in MDA-MB-231 cells in a dose-dependent manner, as measured by Caspase 3/7 activity (Additional file 6: Figure S2).

### Cell growth inhibition induced by HKMT inhibitors

The IC50 of the dual inhibitor compounds HKMT-1-005 in cell viability (MTT assay) or clonogenic growth assays of MDA-MB-231 cells compared to the starting point for the chemical library BIX-01294, as well as UNC0638 and GSK343, are shown in Table 2. Encouragingly, the dual inhibitors show greater inhibition of cell growth as measured both by cell viability and clonogenic assay compared to the EHMT2- or EZH2-specific inhibitors. EZH2 inhibitors are reported to be particularly effective at inhibiting cell growth of cell lines with mutant EZH2 [11, 12]. Indeed, the DB lymphoma cell line which has an EZH2 mutation (Y646N, according to the COSMIC database [38]) was observed to be particularly sensitive to the EZH2 inhibitor GSK343 (Table 3). Consistent with the hit compounds having gained EZH2 inhibitory activity, DB cells were also found to be sensitive to HKMTI-1-005. GSK343 was found to be less potent on all the other lymphoma lines, which express wild type EZH2, with anti-proliferative effects observed at micromolar concentrations of compound treatment. This included the cell line SUDLH8, which has amplified and highly expressed wild type EZH2 (processed data obtained from the Cancer Cell Line Encyclopedia [39]). Interestingly, SUDLH8 is more sensitive to HKMTI-1-005 than the other lymphoma lines with WT EZH2 (Table 3), suggesting that increased sensitivity to this dual inhibitor will not be dependent on cancer cells carrying activating mutations but perhaps any mechanism of increased dependency on EZH2.

**Table 3:** Cell growth IC50 inhibitors in a panel of cell lines

(A) Lymphoma cell lines						
EZH2 status	WSU-FSCLL	WILL1	DOHH2	SC1	DB	SUDLH8
HKMT-I-005	3.405	5.599	3.257	3.711	<1	<1
GSK343	2.868	17.91	6.151	12.12.	<1	5.11
	W.T.	W.T.	W.T.	W.T.	Mutant Y646N	W.T.
(B) Breast cancer cell lines						
IC50 (μM)	MDA-MB-231	MCF-7	T-47D	BT-474	SkBr3	MCF10a
HKMT-I-005	4.3	7.7	8.5	2.1	7.7	>15

Cell growth IC50s (in μM) for a panel of cell lines, after treatment with HKMT-I-005 or GSK343: lymphoma cell lines (A), derived from cell counting following propidium iodide staining, and breast cancer cell lines (B), derived from MTT assays for cell viability. Mutation status of EZH2 is shown for each of the lymphoma cell lines

The anti-proliferative effect of HKMTI-1-005 on a small panel of breast cancer cell lines was determined, with IC50 values in the range 2–10 μM (Table 3). All of the cancer breast cell lines examined were found to be more sensitive to HKMTI-1-005 compared to a normal breast epithelial cell line MCF10a. The breast cancer cell line BT-474, which is the cell line most sensitive to HKMTI-1-005 treatment, has the highest relative expression of EZH2, as detected by Western analysis (data not shown).

### Hit compounds directly inhibit EZH2 and EHMT1/2 and are substrate competitive inhibitors

We have previously reported the EHMT2 IC50 of HKMTI-1-005, HKMTI-1-011 and HKMTI-1-022 to be 0.10, 3.19 and 0.47 μM, respectively [40]. This data was generated using a scintillation proximity assay (SPA) which monitors the transfer of a tritium-labelled methyl group from [^3^H]*S*-adenosyl-L-methionine (SAM) to a biotinylated-H3 (1–25) peptide substrate, mediated by EHMT2. A comparable PRC2 enzymatic assay was employed here to assess biochemical inhibitory activity of our hits against EZH2. A trimeric PRC2 complex (EZH2:EED:SUZ12) was employed in this assay, along with a biotinylated-H3 (21–44) peptide substrate. This revealed HKMTI-1-005, HKMTI-1-011 and HKMTI-1-022 to have PRC2 IC_50_ values of 24, 12 and 16 μM, respectively, under these assay conditions (see Additional file 7: Figure S3). Since the peptide substrates used in these assays are poor models for the complex and dynamic structure of the chromatin substrate in cells and since the only minimal number of PRC2 proteins (EZH2:EED:SUZ12) required for enzymatically active EZH2 were employed in the PRC2 assay, care should be taken in the over interpretation of this in vitro inhibitory data. Nonetheless, we note that both the EHMT2 and PRC2 biochemical potency is comparable to the inhibitory concentrations employed in our cell-based assays.

Perhaps more importantly, in accordance with our design rationale, mechanism of inhibition studies on representative hit HKMTI-1-005 revealed it to have a well-defined, peptide substrate competitive mechanism of action (see Additional file 8: Figure S4), in contrast to all known EZH2 inhibitory chemotypes. Broad screening of our compound library against PRC2 using this assay revealed the IC_50_ values obtained for all actives to be dependent on peptide substrate concentration (data not shown), further confirming a substrate competitive inhibitory mode for this chemotype.

Finally, a methyltransferase selectivity screen was carried out for the hits on a panel of enzymes including 11 HKMTs, 3 protein arginine methyltransferases (PRMTs) and 1 DNA methyl-transferase (DNMT) (Additional file 9: Figure S5). None of the hits had any significant inhibitory activity against these 15 other methyltransferase targets (up to 100 μM), confirming them to be selective for EZH2 and EHMT1/2. Taken together, these data reveal our hit compounds to be dual EZH2 and EHMT1/2 inhibitors with a substrate competitive mechanism of action.

## Discussion

It is widely accepted that the installation, maintenance and functional output of epigenetic modifications occur in concert via combinatorial sets of modifications. Therefore, removal of a single specific repressive mark may not alone be sufficient for reversal of gene silencing. Elimination of multiple repressive methylation marks may instead be required to re-express a wider spectrum of genes. Given the complexities of epigenetic regulation and cross-talk between epigenetic regulators, the discovery of inhibitors of epigenetic processes that lead to reversal of epigenetic silencing may be more suited to cell-based methods measuring reactivation of a panel of target genes, rather than cell-free assays that use purified components. Through the use of a breast cancer (MDA-MB-231) cell assay based on the re-expression of epigenetically silenced genes, we report the identification of hit compounds that phenocopy the effects of dual EZH2/EHMT2 pharmacological inhibition and dual SiRNA gene knockdown.

The recently reported specific EZH2 inhibitors are all co-factor competitive, the majority of which have converged to a common chemotype (Fig. 2) [10–16]. Conversely, the dual EZH2/EHMT2 inhibitors we here report are substrate competitive. Not only do these represent the first inhibitors uniquely targeting the substrate binding site of EZH2 but also confirm our original hypothesis that the histone-binding sites of certain HKMTs are similar [33], and it is therefore possible to discover dual inhibitors targeting this supposedly divergent pocket. Indeed, the results herein suggest that there are common aspects to the histone-binding pockets of the repressive HKMTs, EZH2 and EHMT1/2, different from other HKMTs. Indeed, our selectivity data suggest EZH2 and EHMT1/2 to be the sole HKMT targets of our hit compounds, as does our cell-based data. It is interesting that small changes to the chemical structure of these molecules endow our hits with dual activity, something not observed for the structurally related UNC0638. Indeed, quinazoline EHMT2 inhibitors UNC0638 [24] [22] and UNC0642 [25] have been previously shown not to significantly inhibit EZH2 in biochemical assays.

Amplification or overexpression of EZH2 has been observed in a wide range of tumour types [3–8]. Furthermore, it has been proposed that epigenetic dysregulation can be a contributing factor to acquired drug resistance [7, 8, 41]. In cancers, the specific signalling mechanisms that lead to rapid tumour cell proliferation or evasion of drug-induced apoptosis may vary from cell to cell. One of the appeals of epigenetic therapies in cancer is that, rather than trying to target each individual signalling aberration, the target is the means of acquiring aberrant signalling. Therefore, it is hoped that such therapies may fare better in a heterogeneous tumour environment than drugs targeting specific signalling proteins. In this light, we highlight the observation that a set of EZH2 target genes derived from siRNA knockdown in MDA-MB-231 cells were systematically upregulated following treatment of MDA-MB-231 cells with HKMTI-1-005, but not a set of EZH2 targets were identified from siRNA knockdown in MCF7 cells. This suggests that the compounds are able to elicit a transcriptional response that is specific to a particular cell line, and thus, represent a means of tailoring the response to the targets that are specifically epigenetically repressed in the cancer cells to be treated. However, this fact additionally suggests that it may be difficult to find generally appropriate pharmacodynamics biomarkers indicative of a cellular response to treatment with the compounds. To address this, we carried out a meta-analysis to identify genes with a consistent upregulation following EZH2 knockdown via siRNA across a panel of 18 cell lines. These genes may reflect useful biomarkers for extending the drug-screening process into a wider range of cancer cell lines. We also highlight a potential biomarker of dual EZH2/EHMT2 inhibition in MDA-MB-231 cells, the gene SPINK1, which shows no effect following transfection of siRNA targeting EZH2, nor siRNA targeting EHMT2, but shows increased expression following dual transfection of siRNAs targeting EZH2 and EHMT2. SPINK1 expression is increased upon treatment with HKMT-I-005, HKMT-I-011 and HKMT-I-022 at multiple doses and time points. SPINK1, a potent protease inhibitor, is also known as pancreatic secretory trypsin inhibitor (PSTI), and mutations in SPINK1 have been associated with chronic pancreatitis [42].

Genome-wide expression analysis revealed that genes upregulated upon treatment with HKMTI-1-005 were more enriched for genes silenced by EZH2 than treatment with either the specific EHMT2 inhibitor UNC0638 or the specific EZH2 inhibitor GSK343. It was interesting to note that the EHMT2 inhibitor UNC0638 seemed to be as effective as the specific EZH2 inhibitor GSK343 in terms of specific upregulation of genes silenced by EZH2. This could in part be explained by the fact that EHMT2 has the capacity to methylate H3K27 [26, 27] and that reversal of epigenetic silencing of certain EZH2 targets is dependent on inhibition of EHMT2 [29]. Alternatively, it could be due to differences in the kinetics of the inhibitors that act through different mechanisms, and the fact that genome-wide expression analysis was only carried out within a limited time window.

We also note that the effects observed on gene expression, chromatin marks and global levels of H3K27me3 and H3K9me3 occur within 24–72 h, while some previously reported EZH2 inhibitors only show pharmacodynamic effects at later time points [10, 12, 14–16]. There may be many reasons for these differences, including the mechanism of action of the dual inhibitors, as well as their effects on mRNA levels of EZH2 and the H3K27 demethylase JMJD3. However, it should be noted that the kinetics of effects on gene expression we observe with the dual inhibitors are similar to the kinetics of effects on gene expression we observe with double siRNA knockdown of EZH2 and EHMT2. Also, we observe less effect of GSK343 on H3K27me3 after a 48-h treatment of MDA-MB-231 cells compared to previous studies in ovarian cell line models after 3 days of treatment [43]. This lower activity on H3K27me3 could be cell specific or due to a shorter treatment period. The wealth of cellular data accumulated for our hit compounds, HKMTI-1-005 in particular, argue for direct effects on cells at the target H3K27me and H3K9me modifications at doses of drug less than or equivalent to growth inhibitory doses. Such data includes the specific expression of EZH2 target genes, global histone methylation changes by Western analysis and local chromatin changes on responsive genes. We also note the increased sensitivity of the mutant EZH2 DB lymphoma cell line to HKMTI-1-005, in accordance with an EZH2 inhibitory effect. Such cellular biological effects are observed at doses of hit compounds less than the in vitro biochemical IC50 detected for EZH2. We would argue that the cellular activity is a consequence of dual HKMT activity and so extrapolating from single enzyme IC50 values is difficult. Furthermore, since the in vitro biochemical EZH2 activity assay conditions used the minimal number of proteins (EZH2:EED:SUZ12) and a simple peptide substrate, rather than the complex (and dynamic) in vivo target of chromatin, care should be taken in drawing quantitative comparisons with cell-based data.

The hit compounds reported herein represent starting points for the further optimisation of dual EZH2/EHMT2 inhibitors. Indeed, recent reports suggest it is possible to improve the in vivo profile of this compound class [25]. While this scaffold has been extensively pursued for selective EHMT1/2 inhibition, further studies are needed to confirm whether it is possible to simultaneously increase potency against both EZH2 and EHMT1/2 and whether it is possible to engineer EHMT1/2 activity out of this scaffold to identify a selective substrate competitive EZH2 inhibitor. Nonetheless, it will continue to be important to ‘repurpose’ existing HKMT inhibitor chemotypes, in light of the low number of validated HKMT inhibitory chemotypes currently available [16].

## Conclusions

Many cancers show aberrant silencing of gene expression and overexpression of histone methyltransferases, including EZH2 and EHMT1/2. We have shown that combined inhibition of EHMT1/2 and EZH2 increases growth inhibition in tumour cells over inhibition of only EHMT1/2 or EZH2 and results in re-expression of silenced genes. We report the first dual EZH2-EHMT1/2 substrate competitive inhibitors and show that they may have greater activity in tumour cells that overexpress wild type EZH2.

## Methods

### qRT-PCR measurements for cell-based screening

Following compound treatment of MDA-MB-231 for 48 h (in 6-well plates), media was removed and 1.5 ml of TRIzol (Invitrogen) was added directly to lyse cells and RNA was isolated according to the manufacturer’s instructions. Reverse transcription was done using the SuperScript III First-Strand Synthesis System (Invitrogen) according to the manufacturer’s instructions. Each measurement was done in triplicate, and the List of Primers can be found in Additional file 2: Table S1. For normalisation, we have used GAPDH and RNA pol II. Experiments were also done with the ‘Fast Sybr Green Cell-to-CT™-Kit’ according to the manufacturer’s instructions (Applied Biosystem). Fifteen thousand cells per 96 well were plated, and after 24 h, treated with compounds at various concentrations.

### SiRNA experiments

SiRNA experiments were carried out on the MDA-MB-231 cell line using Qiagen reagents, according to the manufacturer’s instructions. In brief, cells were seeded at a density of 1 × 10^5^ cells/6 cm well and treated for 48 h with siRNAs given in Additional file 2: Table S2.

### Chromatin immunoprecipitation (ChIP-PCR) assay

ChIP was accomplished using Dynabeads Protein A (Invitrogen) according to [44], except that following the Chelex-DNA purification, an additional purification with QIAquick PCR Purification Kit (Qiagen) was carried out; here, the ChIP-products were eluted in 50 μl and for subsequent qPCR measurements (as described above). The list of Primers can be found in Additional file 2: Table S3. Results were calculated as a fold increase of the no-antibody control and then normalised to GAPDH (active marks) and beta-globin (inactive marks).

### Cell viability assay

For clonogenic assays, cells were treated with compound for 48 h, media were removed and replaced with fresh media for a further 12 days to measure colony formation. For cell viability assays, lymphoma cells were plated at 20,000 cells in 200 μl per well in U-bottom 96 well plates in RPMI medium +20 % FCS. Cells were re-suspended 48 h later, diluted 10-fold in PBS + propidium iodide (PI) and the concentration of PI negative cells was counted using an Attune flow cytometer with autosampler. Breast cancer cells were seeded at a density of 10,000 cells/well in a sterile 96 clear-well plate with 150 μl of DMEM (+10 % FCS and 2 mM L-Glutamine). Each compound treatment was performed in triplicate for 72 h at concentrations of 100 nM and 1, 5, 10 and 50 μM in 100 μl of full-medium. After 72 h, 20 μl of MTT solution (3 mg of MTT Formazan, Sigma/1 ml PBS) was added to the medium and incubated for 4 h at 37 °C in a CO_2_ incubator. The MTT-product was solubilised with 100 μl DMSO, and for 1 h, incubated in the dark at room temperature. The optical density was read at 570 nm with PHERAstar.

### Westerns

MDA-MB-231 cells seeded in 6 well plates at a cell density of 3 x 10^5^ were treated with HKMTI-1-005 (1–7.5uM) for 48 h. Following lysis in Triton Extraction Buffer (TEB, PBS containing 0.5 % Triton ×100 (v/v), 1/1000 protease inhibitor), nuclei were re-suspended in 0.2 N HCL at a density of 4 x 10^7^ nuclei per millilitre and incubated overnight at 4 °C to acid-extract the histones, before being centrifuged at 6500*g* for 10 min at 4 °C. Protein concentration was determined using the Bradford assay. H3K27me3, H3K9me3, H3K9me2, H3K9me and total H3 protein expression levels in the histone extract samples were determined using Western blot analysis using H3K27me3 (1:1000; Abcam), H3K9me3 (1:1000; Abcam), H3K9me (1:1000) and H3 (1:2000; Abcam) antibodies. After washing, the membrane was incubated with a horseradish peroxidase-labelled secondary antibody (1 h, room temperature). The membrane was incubated for 1 min with 5 mL of Pierce ECL Western blotting substrate (Thermo Scientific). Images were captured using Konica Minolta SRX101A Tabletop X-Ray film processor.

### Gene expression microarrays

Agilent 80k two-colour microarrays were used to profile gene expression changes induced by treatment with drug compounds in MDA-MB-231 cells, both at 24 and 48 h. In the initial microarray experiment, three replicates were used for each drug, time combination, and in the validation study, four replicates were used. A separate untreated control sample was used for comparison with each replicate. Sample labelling, array hybridization and scanning were performed by Oxford Gene Technologies, according to manufacturer’s instructions. Feature extracted files were imported into GeneSpring (Agilent), and data was normalised to produce log2 ratios of treated/untreated for each replicate of each drug, time combination.

### Statistical analysis

#### Differential expression

Normalised log2 gene expression ratios were analysed using LIMMA [45] to obtain empirical Bayes-moderated *t* statistics for differential expression across the replicates for each drug treatment. After multiple testing adjustments by the Benjamini-Hochberg method, *p* < 0.1 was used to denote significant differential expression in the initial microarray experiment and *p* < 0.05 in the validation experiment.

#### Enrichment analysis

A list of EZH2 targets in MDA-MB-231 cells were taken from [35]. Statistical significance of systematic upregulation or downregulation of these targets was evaluated using the ‘GeneSetTest’ method from the Bioconductor package *limma*. The same method was used to evaluate systematic up- or downregulation of pathways as annotated in ConsensusPathDB [36]. Further analysis was performed using DAVID [46] for exploration of functional annotation enrichments.

### Identification of a set of consensus EZH2-suppressed genes via meta-analysis

A meta-analysis of 18 microarray experiments were carried out as described in ‘Supplementary Methods’, resulting in the list of consensus EZH2 target genes given in Additional file 3: Table S4.

## Additional files

Additional file 1: Figure S1. Global effect of combination HKMT inhibitor treatment on H3K27me3/H3K9me3. Additional file 2:
**Supplementary Methods and Supplementary Tables.**
Additional file 3: Table S4. Described lists of EZH2 targets, including a set of consensus EZH2-suppressed genes. Additional file 4: Table S5. List of study accession numbers for EZH2 target meta-analysis. Additional file 5: Table S6. Systematic pathway up/downregulation following treatment. Additional file 6: Figure S2. Induction of apoptosis in breast cancer cells by compound treatment. Additional file 7: Figure S3. IC_50_ determination for PRC2 inhibitors. Additional file 8: Figure S4. Mechanism of PRC2 inhibition by HKMTI-1-005. Additional file 9: Figure S5. Selectivity of HKMTI-1-005, HKMT1-011 and HKMT-022.
