# Supplementary figures and images for: Dual EZH2 and EHMT2 histone methyltransferase inhibition increases biological efficacy in breast cancer cells
Source: Clin Epigenetics. 2015 Aug 21;7(1):84. doi: 10.1186/s13148-015-0118-9 (PMC4545913; doi:10.1186/s13148-015-0118-9)

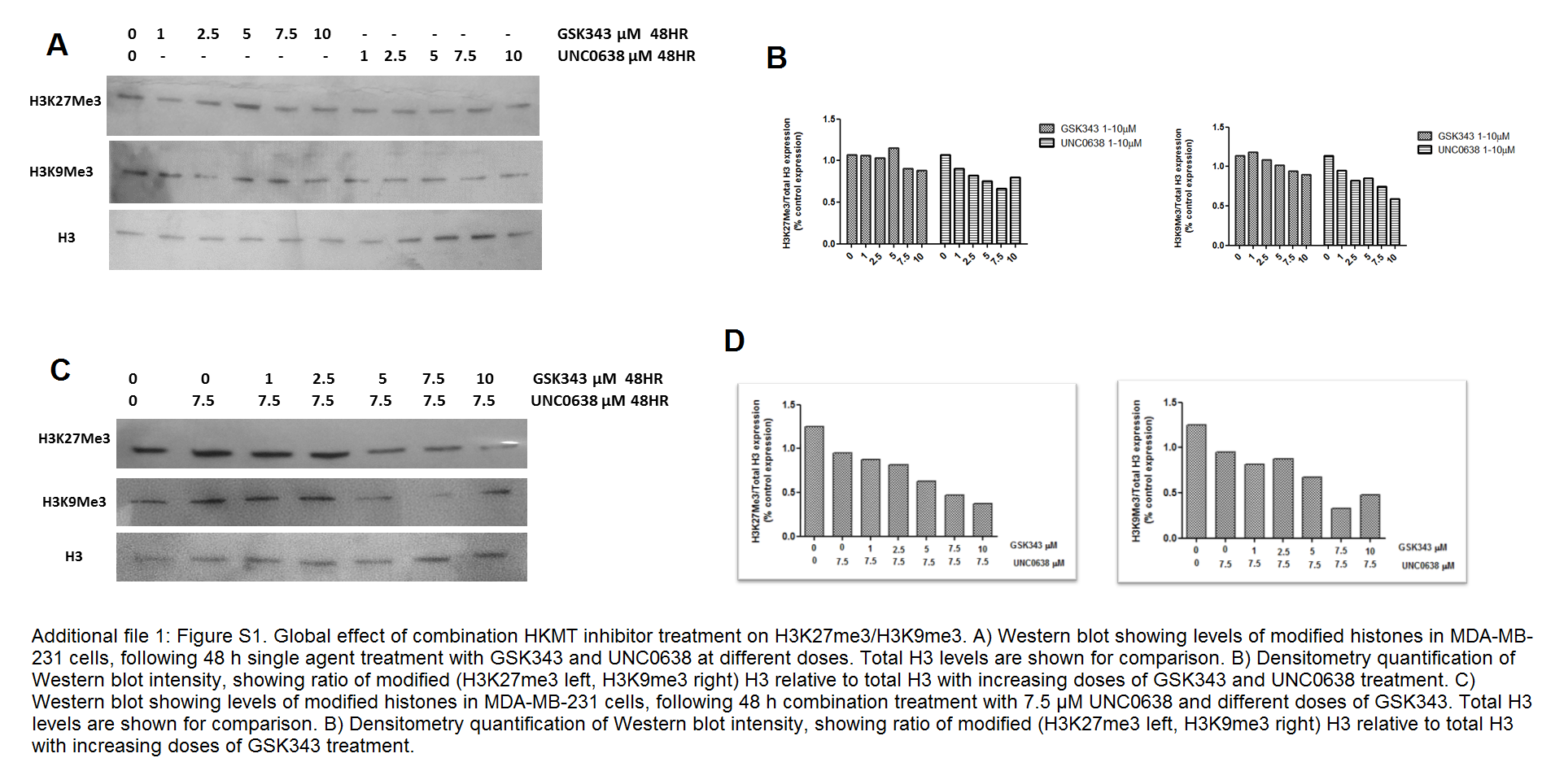

Supplement: Additional file 1: Figure S1. — Global effect of combination HKMT inhibitor treatment on H3K27me3/H3K9me3. [file 13148_2015_118_MOESM1_ESM.png]

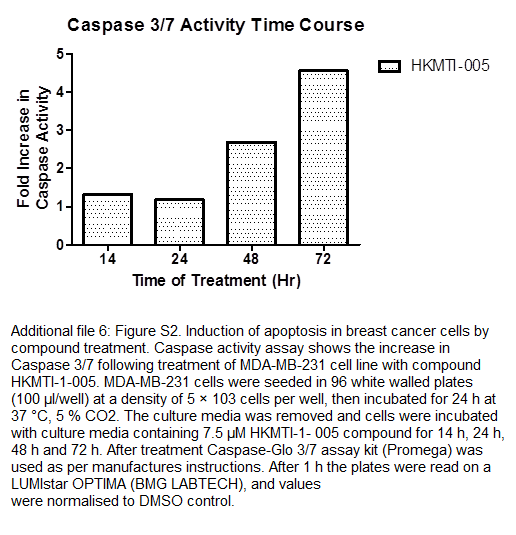

Supplement: Additional file 6: Figure S2. — Induction of apoptosis in breast cancer cells by compound treatment. [file 13148_2015_118_MOESM6_ESM.tiff]

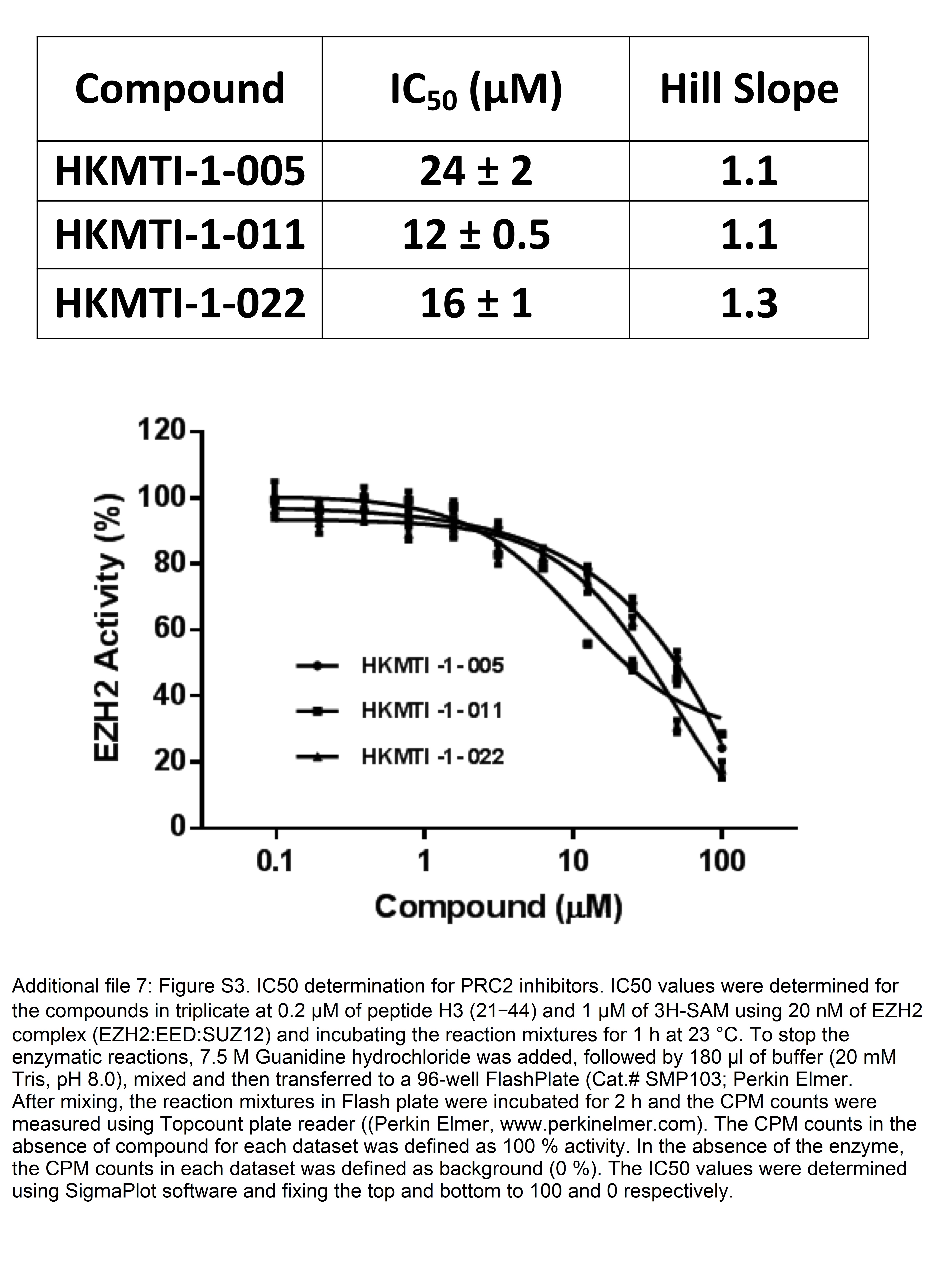

Supplement: Additional file 7: Figure S3. — IC50 determination for PRC2 inhibitors. [file 13148_2015_118_MOESM7_ESM.jpeg]

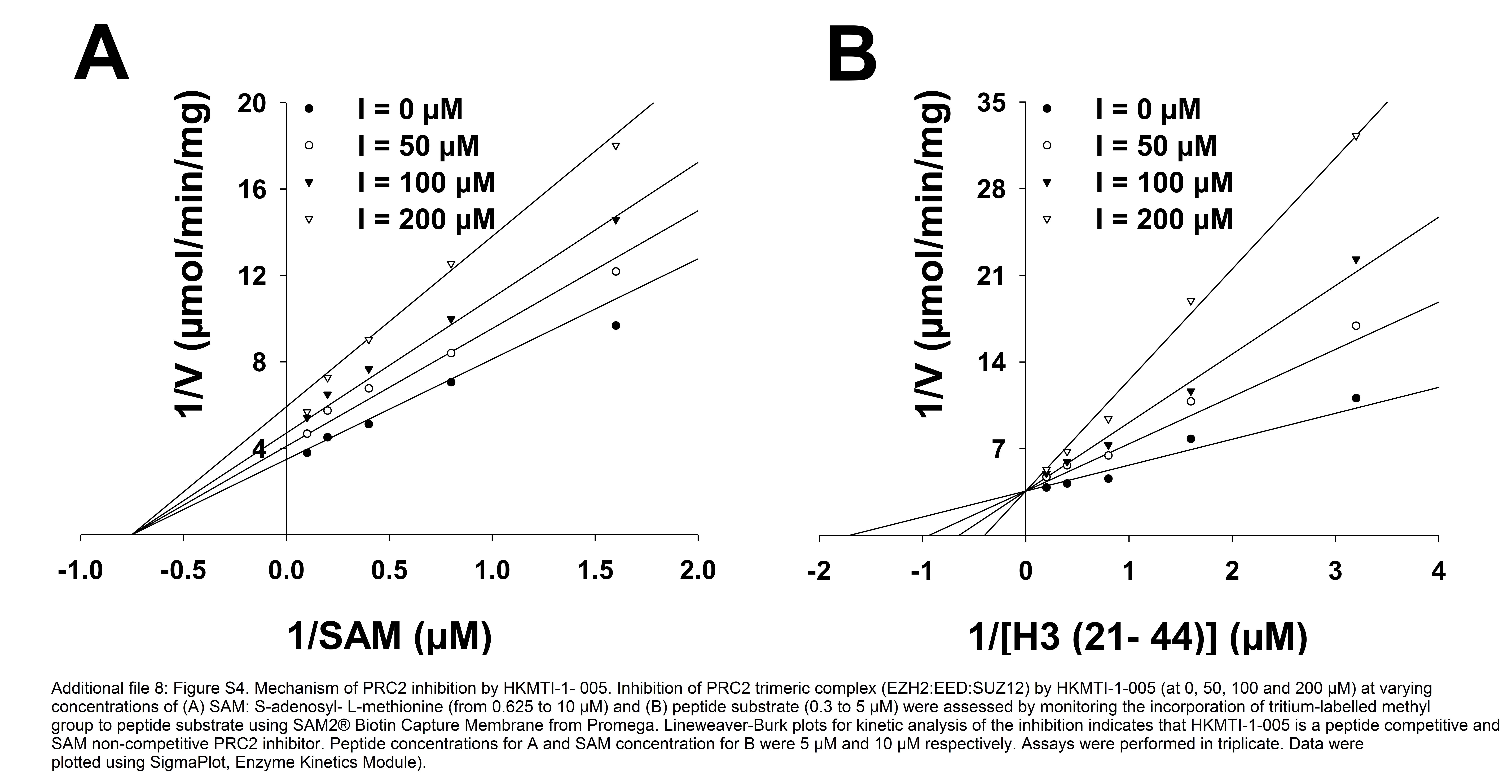

Supplement: Additional file 8: Figure S4. — Mechanism of PRC2 inhibition by HKMTI-1-005. [file 13148_2015_118_MOESM8_ESM.jpeg]

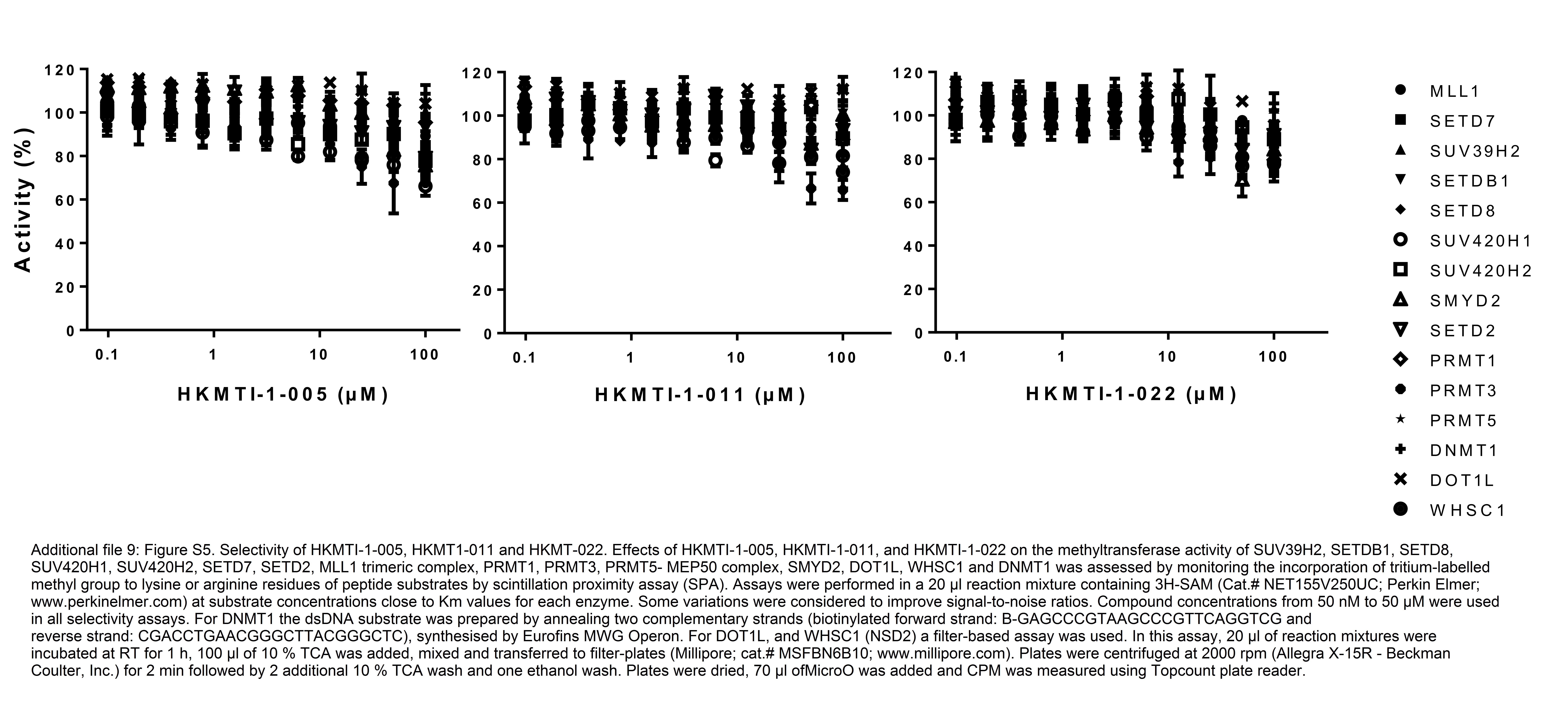

Supplement: Additional file 9: Figure S5. — Selectivity of HKMTI-1-005, HKMT1-011 and HKMT-022. [file 13148_2015_118_MOESM9_ESM.jpeg]
