# Supplementary material for: Dual EZH2 and EHMT2 histone methyltransferase inhibition increases biological efficacy in breast cancer cells
Source: Clin Epigenetics. 2015 Aug 21;7(1):84. doi: 10.1186/s13148-015-0118-9 (PMC4545913; doi:10.1186/s13148-015-0118-9)
Supplement: Additional file 2: — Supplementary Methods and Supplementary Tables. [file 13148_2015_118_MOESM2_ESM.docx]

Supplementary Methods and Tables

**Supplementary Table S1: Primers for quantitative PCR**

For qRT-PCR measurements the 2x iQ SYBR Green Supermix (Bio-Rad), 200nM Primers and 0.4μl of cDNA /per 20μl reaction was used. The measurement was done in low-white 96-well plates (Bio-Rad) on a CFX96 Real-time System/C1000 Thermal Cycler (Bio-Rad) with the following protocol: 95°C for 3’; 95°C for 10’’, 56°C for 10’’, 72°C for 30’’ 42 cycles followed by a melting curve from 72°C to 95°C.

| Name of gene | Forward | Reverse | Product | Pubmed REF |
| --- | --- | --- | --- | --- |
| GAPDH_1 | CCTGTTCGACAGTCAGCCG | CGACCAAATCCGTTGACTCC | 101bp | 12615716 |
| GAPDH_2 | CCCCTTCATTGACCTCAACTACAT | CGCTCCTGGAAGATGGTGA | 135bp | PMC2517635 |
| KRT17 | CAACACTGAGCTGGAGGTGA | GGTGGCTGTGAGGATCTTGT | 124bp |  |
| FBXO32 | TGTTGCAGCCAAGAAGAGAA | CAATATCCATGGCGCTCTTT | 120bp | Primer 3 |
| JMJD3 | CCTCGAAATCCCATCACAGT | GTGCCTGTCAGATCCCAGTT |  |  |
| EZH2 | AGTGTGACCCTGACCTCTGT | AGATGGTGCCAGCAATAGAT | 122bp | RTPrimerDB probe ID: 4521 |
| SPINK1 | GTAAGTGCGGTGCAGTTTT | CCTTGGCCCTGTTGAGTCTA | 101bp | Primer 3 |

**Supplementary Table S2: siRNA sequences**

HiPerfect, Optimem and 50nM of EHMT2/G9a (SI00091189 HS_BAT8 1, SI03083241 HS_EHMT2) and EZH2 (SI00063973 HS_EZH2 4, SI02665166 HS_EZH2 7) siRNA were used for transfections according to the manufactures instructions.

| **Product Name** | **Target Sequence** | **Manufacturer/Catalogue#** |
| --- | --- | --- |
| G9a( HS_BAT8_ 1) | ATCGAGGTGATCCGCATGCTA | QIAGEN  SI00091189 |
| G9a( HS_EHMT2_ 1) | CCTCTTCGACTTAGACAACAA | QIAGEN  SI03083241 |
| EZH2(HS_EZH2_ 4 ) | TTCGAGCTCCTCTGAAGCAAA | QIAGEN  SI00063973 |
| EZH2(HS_EZH2 _7) | AACCATGTTTACAACTATCAA | QIAGEN  SI02665166 |

**Supplementary Table S3: Primers for ChIP-PCR:**

| Name | Forward | reverse |
| --- | --- | --- |
| ChIP_FBXO32 | TTTTCTCCACTCCCAACCTG | GCCCCTTAGCTGTCACTAACC |
| ChIP_KRT17 | TGGCATTGATGAGTGAGAGG | AGCCGAGAGACATTCCTCAA |
| ChIP_GAPDH | CACCGTCAAGGCTGAGAACG | ATACCCAAGGGAGCCACACC |
| ChIP_Beta-globin | GCTGGTGGTCTACCCTTGGA | AGGTTGTCCAGGTGAGCCAG |
| ChIP_SPINK1 | TTGCCTAGTGTGTGATGCAA | GCGAAATCCATGCCTTCTAA |

**Supplementary Table S4: Described lists of EZH2 targets, including a set of consensus EZH2-suppressed genes**

Three lists of EZH2 targets used in this study are provided in Table S4. First column, ‘MDA-MB-231 EZH2 targets (Lee et al)’ was obtained from Ref #35 of the main document. Second column, ‘MCF7 EZH2 targets (Tan et al)’ was obtained from Ref #30 of the main document. The third column, ‘Consensus EZH2 targets (meta-analysis)’, was generated as follows:

Raw data for 18 microarray experiments profiling RNA from EZH2 RNAi treated cells were downloaded from Gene Expression Omnibus ([1](#_ENREF_1)) and processed individually to minimise cross-array platform bias. A list of the study accession numbers is provided in Supplementary Table S5. For each study, a linear regression model was fit to probe intensity values using the R package *limma (*[*2*](#_ENREF_2)*)*. This generated empirical-Bayes moderated t-statistics for the EZH2 RNAi induced differential expression. To reconcile cross-platform probe IDs, HGNC gene symbols were used to identify genes. For genes with multiple probes on an array platform, the most statistically significant differentially expressed probe was used and all others discounted. Three meta-analysis approaches were taken to find genes with consistent upregulation following knock-down of EZH2: Fisher’s method of combining P-values, the Rank Product method ([3](#_ENREF_3)), and a Random Effects Model ([4](#_ENREF_4)). 88 genes were found to show significant consistent upregulation (p<0.05) according to all three methods. This list of genes, which was used to reflect a consensus set of universally (or at least generally) EZH2-suppressed targets, is provided in SupplementaryTableS4.xslx.

**Supplementary Table S5: List of study accession numbers for EZH2 target meta-analysis**

File SupplementaryTableS5.xlsx describes the studies used for the meta-analysis performed to obtain a set of consensus EZH2-suppressed targets. This table provides Gene Expression Omnibus accession numbers and PubMed IDs for the studies, and lists cell lines the siRNA treatment was performed on, the microarray platform used, and the number of replicates for treatment and control samples in each study.

**Supplementary Table S6: Systematic pathway up/down-regulation following treatment**

Systematic up- or down-regulation of pathways was evaluated using the Wilcoxon Rank-Sum test, as implemented by the ‘geneSetTest’ function in the Bioconductor package *limma,* on the empirical Bayes moderated t-statistics of differential expression calculated using *limma* across the 3 replicates from each drug treatment condition. Resulting Benjamini-Hochberg adjusted p-values, for 24hr treatment of GSK343, HKMTI-1-005 (at 7.5µM) and UNC0638 (at 7.5µM) are provided in file SupplementaryTableS6.xlsx.

**Compound batch data:**

The following batches of the hit compounds were used in this study:

**HKMTI-1-005**

TG3-178-2 (synthesized 30/09/2008); NS-011 (synthesized 7/5/2011); NS-080 (synthesized 23/4/12); JC-087 (HCl salt formulation, synthesized 1/10/2012); NS-382 (synthesized 22/08/14)

**HKMTI-1-011**

TG3-214-1 (synthesized 13/11/2008); NS-014 (synthesized 20/6/11); NS-081 (synthesized 26/4/12)

**HKMTI-1-022**

TG3-179-1 (synthesized 20/10/2008); NS-015 (synthesized 28/6/11); NS-082 (synthesized 26/4/12)

**Compound characterization data:**

^1^H NMR (400 MHz, CDCl_3_): δ 7.34-7.27 (m, 5H), 6.90 (s, 1H), 6.70 (s, 1H), 5.02 (d, *J* = 7.2 Hz, 1H), 4.19–4.09 (m, 1H), 3.94 (s, 3H), 3.92 (s, 3H), 3.86 (m, 4H), 3.55 (s, 2H), 2.91 (m, 2H), 2.49 (m, 4H), 2.34 (s, 3H), 2.21 (m, 2H) 2.15 (m, 2H), 1.62 (qd, *J* = 2.9, 11.5 Hz, 2H); ^13^C (100 MHz, CDCl_3_): δ 158.2, 157.8 (br), 154.5, 147.4 (br), 145.7, 137.8, 129.2 (2C), 128.3 (2C), 127.2, 105.3, 103.0, 100.9, 62.9, 56.4, 56.1, 55.02 (2C), 52.4 (2C), 48.3, 46.1, 44.1 (2C), 31.9 (2C); LCMS: R_t_ = 2.69 min; HRMS (ESI) (*m/z*): [M+H]^+^ calcd. for C_27_H_37_N_6_O_2_, 477.2978; found: 477.2970.

^1^H NMR (400 MHz, CDCl_3_): δ 7.34-7.24 (m, 5H), 6.90 (br s, 1H), 6.68 (s, 1H), 4.95 (br s, 1H), 4.19–4.09 (m, 1H), 3.95 (s, 3H), 3.93 (s, 3H), 3.80 (m, 4H), 3.55 (s, 2H), 2.91 (m, 2H), 2.21 (m, 2H) 2.15 (m, 2H), 1.64 (m, 8H); ^13^C (100 MHz, CDCl_3_): δ 160.3, 157.6, 152.4, 149.2, 137.4, 132.4 (2C), 131.2, 130.7, 130.3 (2C), 105.5, 103.5, 99.9, 61.7, 57.4, 56.9, 52.6 (2C), 48.2, 47.4 (2C), 29.4 (2C), 26.7 (2C), 25.1; LCMS: R_t_ = 3.86 min; HRMS (ESI) (*m/z*): [M+H]^+^ calcd. for C_27_H_36_N_5_O_2_, 462.2868; found: 462.2855

^1^H NMR (400 MHz, MeOD): δ 8.16 (m, 1H), 7.76 (br s, 1H), 7.62 (m, 3H), 7.51 (m, 3H), 7.19 (br s, 1H), 6.92 (m, 1H), 6.75 (m, 1H), 4.62 (m, 1H), 4.34 (br s, 2H), 4.04 (m, 4H), 3.98 (s, 3H), 3.96 (s, 3H), 3.77 (m, 4H), 3.57 (br *app*-d, *J* = 12.4 Hz, 2H), 3.30 (m, 2H), 2.34 (br *app*-d, *J* = 12.6 Hz, 2H), 2.17 (m, 2H); LCMS: R_t_ = 3.22 min; HRMS (ESI) (*m/z*): [M+H]^+^ calcd. for C_31_H_38_N_7_O_2_, 540.3087; found: 540.3077

Purification was achieved by silica, amino-silica or acidic alumina column chromatography.

LCMS gradient: From 95% A:5% B  to  5% A:95% B over 10 minutes, where A is water (0.1% formic acid), and B is acetonitrile.

**1. Edgar R, Domrachev M, & Lash A (2002) Gene expression omnibus: NCBI gene expression and hybridization array data repository. *Nucleic Acids Res* 30(1):207-210.**

**2. Smyth GK (2004) Linear models and empirical bayes methods for assessing differential expression in microarray experiments. *Statistical Applications in Genetics and Molecular Biology* 3(1):3.**

**3. Breitling R, Armengaud P, Amtmann A, & Herzyk P (2004) Rank products: a simple, yet powerful, new method to detect differentially regulated genes in replicated microarray experiments. *FEBS Letters* 573(1–3):83-92.**

**4. Choi JK, Yu U, Kim S, & Yoo OJ (2003) Combining multiple microarray studies and modeling interstudy variation. *Bioinformatics* 19(suppl 1):i84-i90.**
